# Developing a Psychiatry Residency Program in a Low- and Middle-Income Country: Botswana’s Experience

**DOI:** 10.1177/23821205241310784

**Published:** 2025-01-15

**Authors:** Philip R. Opondo, Anthony A. Olashore, James O. Ayugi, Masego B. Kebaetse, Keneilwe Molebatsi

**Affiliations:** 1 Department of Psychiatry, Faculty of Medicine, 54547 University of Botswana, Gaborone, Botswana; 2 Mahalapye District Hospital, Ministry of Health, Gaborone, Botswana; 3 Department of Medical Education, Faculty of Medicine, 54547 University of Botswana, Gaborone, Botswana

**Keywords:** psychiatry, residency training program, curriculum development, Botswana

## Abstract

Like many other low- and middle-income countries, Botswana has struggled to address the shortage of doctors, particularly specialists. In 2009, the country’s first medical school offering an undergraduate medical program was established. A needs and feasibility assessment was conducted with relevant stakeholders to explore the need for specialty training programs in all medical school departments. As a result, three residency programs were established a year after the undergraduate program, but psychiatry was not included in this first crop of residency programs. Existing strengths in the university and healthcare systems were leveraged to successfully establish a four-year Master of Medicine residency program in psychiatry, which began in January 2020. The program aims to produce psychiatrists who are familiar with the local mental health needs. The first batch of students completed their training in December 2023. This paper reflects on and describes the development of a psychiatry residency program in Botswana and contributes a process grounded in educational program development models and educational theories that others can utilize. Botswana’s experience may be helpful to other low- and middle-income countries, particularly in sub-Saharan Africa, that want to establish and run a locally developed residency program in psychiatry.

## Introduction

Multiple studies have highlighted the shortage of healthcare workers at various levels, especially medical specialists, in Africa.^1–3^ This shortage has resulted mainly from the migration of healthcare workers and the few postgraduate training programs in sub-Saharan Africa.^4^ Like many other sub-Saharan countries, Botswana initially dealt with this shortage by sending its citizens abroad for medical training.^5^ However, many such doctors sent to developed countries for training tended not to return to their countries, thus negating the aim of increasing the number of doctors in their countries of origin.^6–8^ This reality partly contributed to a marked increase in medical schools in sub-Saharan Africa, including private ones, which hoped to increase the number of locally trained doctors.^9^

Despite the increased availability of undergraduate training, many doctors who want to specialize still have to go abroad due to limited postgraduate training capacity.^10^ The lack of specialized training programs is more pronounced in some specialties like psychiatry. This has led to a significant shortage of psychiatrists in LMICs, although mental illness and substance abuse disorders account for four of the top ten causes of health-related disability.^11^ A survey of psychiatric training across the world showed that less than 50% of African countries had psychiatric training programs.^2^ By 2020, Botswana had only 13 psychiatrists (only four of whom were locals), serving a population of 2.3 million, with most of the psychiatric care being provided by psychiatric nurses.^12^ This number has since increased to 17 psychiatrists by September 2024, with the number of locals having increased to 10, mainly due to our program’s first cohort of graduates.

Botswana, an upper middle-income country, has an area of 582,000 km^2^ and a population of slightly over 2.6 million, with a per capita GDP of approximately $20.36 in 2022.^13^ The country's healthcare system follows a primary healthcare model and is organized hierarchically, with the national referral hospitals at the apex and health posts at the bottom of the pyramid. There is one national psychiatric referral hospital, Sbrana Psychiatric Hospital (SPH), with a capacity of 300 beds. Although trained mental health personnel, including psychiatrists, are needed at all healthcare system levels, by 2020, there were very few training opportunities for such personnel. Most of the training was for psychiatric nurses, but there was no local postgraduate psychiatric training program for medical doctors. It has been observed that psychiatric training is best carried out in-country to address the mental health realities of a country appropriately,^2^ and there is evidence of the success of programs that equip their residents to practice in their environment.^6,14^

Faced with the reality that a substantial number of its citizens were sent abroad for medical training and did not come back, Botswana decided to start in-country training of its doctors, leading to the setting up of a medical school in 2009.^5^ The following year (2010), the medical school started a sequenced introduction of residency programs in pediatrics, pathology, public health, internal medicine, family medicine, anesthesia, and emergency medicine. The eventual aim was to have all departments in the faculty develop residency programs, and due to the country’s lack of psychiatrists, it was decided to start a residency program in psychiatry.

The budding literature on the development of psychiatry programs from the African context tends to describe the collaboration between high-resourced nations and low-resourced partners that made the development of the residency program possible, especially in Liberia,^15,16^ Somaliland,^17^ and Ethiopia.^18^ Although countries make a case for their psychiatry programs, for example, Liberia had only two psychiatrists in 2018, there seem to be limited micro-level explicit descriptions of how programs were developed beyond making partnerships work. Also, there appears to be limited sharing of learning outcomes, program structure, and the theoretical underpinning driving program design and delivery.

This paper adds to the sparse literature on developing psychiatry programs in sub-Saharan Africa by using curriculum development models and drawing from educational theory to reflect and describe the development of a psychiatry residency program in Botswana.

## Our Program Development Process

The program development was driven by the Department of Psychiatry, University of Botswana, which comprised five staff members (four psychiatrists and one clinical psychologist). To guide the development of our program, we used the ADDIE model and integrated Kern's six-steps^19^ and the Fink Integrated Course Design model^20^ to elaborate on the elements of the ADDIE. This provided a systematic approach to our curriculum development process, allowing us to be sensitive to our context and learners’ needs and enabling us to ensure constructive alignment of learning outcomes, learning methods, and assessment strategies. The interrelated iterative phases of our program development were grounded on the phases of the ADDIE (analysis, design, development, implementation, and evaluation) and are described below.

## Analysis Phase

In the analysis phase, we focused on the needs assessment and integrated Kern's problem identification and needs assessment^19^ and Fink's situational analysis.^20^ The needs assessment is essential to ensure that the program developed is contextually relevant, sensitive to the needs of the learners, and overall fit for purpose. The needs assessment, especially stakeholder engagement, draws from the humanistic perspective of curriculum development, of giving voice to and ensuring that the ensuing program is grounded in the felt needs of those affected.^21^ The macro-level (national) aspect focused on determining the need for the training program, and the micro-level (program) aspect focused on a targeted contextual and situational analysis.

A national Task Force on Medical Education conducted the macro-level national needs assessment in the 1990s. It was becoming unsustainable for the Botswana Government to train doctors abroad who were not returning home to serve.^22,23^ As such, the task force was convened to study the feasibility of establishing a medical school,^8^ which was ultimately established and enrolled its inaugural cohort in 2009. In addition to undergraduate medical training, the task force recommended a phased approach to starting residency programs to grow a national crop of specialists, considering the low numbers of specialists in the country, the difficulty attracting enough specialists, and the few training positions in the region (Southern Africa). These residency programs were also seen as a retention strategy for the expected growing number of general practitioners. It was determined that applicants to the program would have a first degree in Medicine from a recognized university, have completed at least two years of clinical practice (including a minimum of 12 months in a recognized supervised internship program), and be registered or eligible for registration with the Botswana Health Professions Council (BHPC).

Having established the need for residency programs in Botswana, including psychiatry, the Department of Psychiatry conducted a targeted, micro-level needs assessment in 2019 to kickstart the development of the psychiatry residency program. Even after 20 years from the national needs assessment, and despite a relatively high burden of mental disorders at the time (31,233 national mental health outpatient consultations),^24^ Botswana still had a low number of psychiatrists (13) in private and public practice; four of those in public service were at the medical school. The targeted needs assessment involved consultations with key stakeholders, including the Ministry of Health, the Botswana Health Professions Council, the Centre for Academic Development (CAD) of the University, the Department of Medical Education, other departments in the Faculty of Medicine that already had residency programs, and public and private sector psychiatry specialists. We also engaged with community members - health professionals, the lay public, and newly qualified doctors from the medical school (potential students for the program).

The stakeholder meetings confirmed the need for training more mental health professionals, given the fact that mental health problems were common in the country. In particular, the doctors interested in psychiatry expressed the need to train locally to avoid undue disruption to their personal lives occasioned by their going to train abroad. A localized psychiatric training program would offer a cost-effective way of meeting psychiatrists’ needs and help fill some gaps in locally generated research knowledge. They also felt that the unique sociocultural factors of the local populations were not adequately covered in the literature, so further research was needed in those areas. The program would also enhance some of the existing programs at the University by providing courses for residents in related disciplines, such as internal medicine, pediatrics, and family medicine, who need to take some modules in mental health. Other students taking psychology and social science courses would also benefit from modules in behavioral sciences offered by the proposed psychiatry program.

In addition to stakeholder meetings, the department also engaged in internal SWOT and situational analyses, examining our learners, teaching staff, and teaching platform to assess strengths, gaps, and possible remediations consistent with the Fink Integrated Course Design model.^20^ The analyses revealed that we were starting with certain program development advantages. First, an existing department of psychiatry within an established university allowed us to leverage available university resources. For instance, the University had a mature and robust process for developing and approving new academic programs, which required us to assess critical factors for establishing our program: curriculum design, library resources, financial resources, academic services, and processes for interfacing and aligning with regulatory bodies. Second, we had access to a 300-bed psychiatric referral hospital, where the university had already set up the necessary infrastructure for teaching and learning, such as the library and onsite librarians, teaching rooms, and internet access.^5,25^

Our main weakness was that the department had only five academic staff members already teaching in a growing undergraduate program. Consequently, the cohort size would be limited to five residents per year. We also faced challenges regarding staff recruitment and retention similar to those faced by pioneer residency programs in the Faculty of Medicine, such as the inability to attract high-quality academic staff, including subspecialists, and a high attrition rate of academic staff.^5,26^ We had to consider other means to address these potential shortcomings, such as engaging in regional and international collaborations to complement the existing teaching staff.

## Design Phase

In the design phase, we focused on developing program learning outcomes and course structure, as well as determining and selecting instructional and assessment activities based on the program learning outcomes. This involved integrating Kern's objectives and educational activities,^19^ Fink's learning goals, teaching/learning activities, and feedback and assessment.^20^ This phase drew from backward design,^27^ behaviorism,^28^ cognitive theory,^28^ and constructivist theory.^28^ Backward design and behaviorism challenged us to think critically and articulate clear, measurable outcomes from which learning and assessment activities would be determined. Constructivist and cognitive theories guided the articulation of outcomes supporting activities encouraging collaborative learning, problem-solving, personal exploration, and higher-order thinking. Biggs's constructive alignment^29,30^ principle was the grounding frame that challenged us to think critically regarding the alignment of outcomes, instructional and learning methods, and assessment and feedback activities.

Having established the need for the program, its situational factors, and broad learning goals, extensive benchmarking was conducted against qualifications offered by reputable institutions within the region and beyond. This enabled us to appreciate what is typical of this level and type of qualification concerning graduate profiling, scope, and depth of content. To ensure a good standard of regional and international comparability, we discussed our draft curriculum with academics from other universities, compared theirs with our proposed curriculum, and incorporated their comments and recommendations where necessary.

Psychiatric residency programs worldwide differ in various aspects, such as the scope of their curriculum content, duration of the training, prerequisites for entry, and accreditation criteria.^31^ Our program was modeled on the World Federation for Medical Education guidelines^32^ and other sources. We mapped and aligned our outcomes to the Botswana Health Professions Council competencies and the Botswana Qualifications Authority guidelines. We also drew on our experiences developing our undergraduate psychiatry program.^33^ Faculty of Medicine residency programs already accredited on the national qualification framework provided another source of comparison.

### Program outcomes

Table 1 describes our graduate outcomes and assessment indicators. Overall, there were seven core outcomes for the program: (a) assess patients with behavior and emotional disorders to make a diagnosis and differential diagnoses; (b) manage patients presenting with emotional and behavioral problems in keeping with the latest evidence-based guidelines; (c) evaluate the quality and utility of newly acquired information to make informed decisions; (d) demonstrate a high degree of professionalism in clinical care and relationships with colleagues in the work environment; (e) advise patients and the broader community on matters pertaining to health promotion and disease prevention; (f) apply advanced knowledge and skills in the articulation of mental health issues and training of relevant stakeholders; and (g) engage in mental health research to address identified relevant needs.

**Table 1.** Graduate outcomes and assessment criteria.

Learning outcomes	Assessment criteria
1 Assess patients with behavior and emotional disorders to make a diagnosis and differential diagnoses.	1.1 Compile a comprehensive psychosocial and psychiatric history and conduct a mental state examination. 1.2 Perform appropriate neuropsychiatric tests. 1.3 Present the clinical findings and make a case, including the diagnosis and differential diagnoses to aid in managing patients with mental health problems.
2 Manage patients presenting with emotional and behavioral problems in keeping with the latest evidence-based guidelines.	2.1 Request and interpret appropriate laboratory or radiological investigations of patients with psychiatric disorders. 2.2 Initiate appropriate pharmacological treatment for patients with mental health problems. 2.3 Conduct psychotherapy sessions with patients presenting with psychosocial problems when needed. 2.4 Communicate treatment information to the patient and relatives. 2.4 Counsel relatives of patients with mental health problems. 2.5 Communicate effectively with lay persons, patients, and the families of the mentally ill. 2.6 Communicate relevant information on mental health issues effectively in professional situations, demonstrating care, respect, and ability to work in a team.
3 Evaluate the quality and utility of newly acquired information to make informed decisions.	3.1 Use information technology in searching for relevant mental health literature. 3.2 Design or develop future mental health care provision. 3.3 Review critically and appraise clinical practice of current and emerging mental health trends. 3.4 Engage in continuous education in fields relevant to Psychiatry.
4 Demonstrate a high degree of professionalism in clinical care and relationships with colleagues in the work environment.	4.1 Demonstrate ethical behavior, leadership, and planning skills to effectively problem-solve and efficiently deliver mental health services. 4.2 Collaborate with people with mental health disorders, other health professionals, and stakeholders in mental health to promote mental health.
5 Advise patients and the broader community on matters pertaining to health promotion and disease prevention.	5.1 Educate and motivate communities to take personal responsibility for their mental health care. 5.2 Advocate for the promotion of mental health at all levels.
6 Apply advanced knowledge and skills in the articulation of mental health issues and training of relevant stakeholders.	6.1 Mentor, train, supervise, and appraise mental health workers at all levels. 6.2 Make appropriate evidence-based recommendations for preventive and therapeutic interventions. 6.3 Attend academic meetings regularly to keep abreast with new trends and practices in the psychiatric field.
7 Engage in mental health research to address identified relevant needs.	7.1 Identify a suitable research topic in the field of psychiatry. 7.2 Conduct a literature search to establish current knowledge concerning the selected topic in psychiatry. 7.3 Design a comprehensive protocol for submission for ethical approval for the research proposal. 7.4 Collect the necessary data. 7.5 Analyze the data to arrive at conclusions. 7.6 Prepare an original research thesis and/or article for publication in a recognized academic journal.

### Program structure

Having articulated the program outcomes, we determined the content areas/courses needed to enable the learners to achieve the intended program outcomes. The program is an eight-semester, four-year, full-time program divided into two equal parts: Part 1 covers the first two years, and Part 2 covers the last two years (Table 2).

**Table 2.** Course sequence and core components of the curriculum.

Part I: Years 1 and 2	Semester I: Communication, Ethics, and Professionalism; Introduction to Psychiatry I (behavioral science and sociocultural psychiatry; human development; clinical neurosciences, clinical psychopathology; mental health problems: classification and practice of psychiatry).
	Semester II: Introduction to Clinical Research; Introduction to Psychiatry II (neuropsychiatric assessment and neuroimaging); Introduction to Medical Literature; Dissertation 1.
	Semester III: Dissertation 2; Intermediate Psychiatry I (clinical psychopharmacology; general adult psychiatry); Principles and Techniques of Medical Education.
	Semester IV: Public Health Principles & Global Health; Dissertation 3; Intermediate Psychiatry II (neuropsychiatry/neurology; treatment modalities in psychiatry: biological and physical therapies).
Part II: Years 3 and 4	Semester V: Dissertation 4; Introduction to Healthcare Management; Intermediate Psychiatry III (addiction/ substance misuse psychiatry; emergency psychiatry).
	Semester VI: Advanced Psychiatry I (child and adolescent psychiatry; psychotherapy; public mental health and community psychiatry).
	Semester VII: Presentation and Defense of Dissertation; Advanced Psychiatry II (learning disability; forensic psychiatry; geriatric psychiatry).
	Semester VIII: Advanced Psychiatry III (consultation-liaison psychiatry)

The first two years (Part I: Years 1 and 2) last for four semesters and are spent on basic and general psychiatric training. Courses include communication, ethics and professionalism, neuroscience, behavioral science, sociocultural psychiatry, psychopathology, psychopharmacology, and other treatment modalities in psychiatry. During these first two years, residents are stationed at the teaching hospital and attached to specialists who supervise them. Part 1 also includes four short (1‒2 weeks) courses common to other Master of Medicine residents in all disciplines in the Faculty of Medicine. These cover ethics, communication, professionalism, and research methods.

The last two years (Part II: Years 3 and 4) are four semesters long and involve rotation through psychiatric subspecialties, such as forensic, child and adolescent, geriatric, psychiatric emergencies, and community psychiatry. The residents are expected to write and present a dissertation of 2500‒10,000 words, which they are prepared for during the research methods courses. They are also encouraged to publish their work.

### Instructional approaches

In determining instructional approaches, we considered the learning outcome and domain of learning and drew from multiple theoretical perspectives, including clinical apprenticeship,^34,35^ situated learning,^36^ experiential learning,^37^ communities of practice,^38^ and self-directed learning.^39^ Clinical apprenticeship is mostly facilitated through outpatient and inpatient clinics, bedside teaching, and ward rounds. Complementary instructional strategies include case presentations and selected topic discussions during morning meetings, monthly multi-disciplinary clinical case conferences, weekly journal clubs, audit projects, morbidity and mortality meetings, monthly research dissertation meetings, tutorial topic presentations, and protected times for self-guided study and practice. These teaching methods enable trainees to increase their theoretical knowledge while developing appropriate clinical skills and competencies.

### Assessment approaches

The residents undergo both formative and summative assessments. Throughout their training, residents prepare a learning portfolio, including a record of clinical rotations/experience and academic activities. They also have a logbook to record case presentations and all other learning activities, which the supervisors sign off on. Eligibility to sit for the examinations depends on completing the required activities in the logbooks.

Summative assessment comprises a mock examination at the end of each semester and an exam at the end of the first two years (Part 1) and the fourth year (Part 2). Both sets of examinations undergo internal and external moderation. Residents must pass the Part 1 exam to proceed to Part 2 of the training. They also must pass their research dissertation and coursework to qualify for the Master of Medicine in Psychiatry degree.

## Development Phase

In the development phase, we focused on developing materials and resources such as course guides, related instructional material, assessment tools, and program policies and guidelines needed to support teaching, learning, and assessment activities. This involved integrating Kern's educational strategies,^19^ as well as Fink's learning goals, teaching/learning activities, feedback and assessment.^20^ and challenged us to ensure all the essential elements are in place for implementation.

### Course guides

We designed a template to ensure the inclusion of essential course elements such as course outcomes, teaching and learning methods, course deliverables, and course schedules in all our course guides.

### Instructional and assessment material

To complement our course guides, we also developed resources such as logbooks to document daily activities and the resident portfolio to stimulate reflective learning by documenting and thinking critically about their training experiences. We also developed checklists and rubrics to ensure clarity in our assessment procedures.

### Program guide

We developed a program guide based on University and faculty policies to ensure residents have the necessary information about the program.

## Implementation phase

In this phase, we focused on implementing and managing essential elements to ensure effective program delivery. This phase involved Kern's implementation^19^ and Fink's view of addressing important details^20^ such as the grading system and orienting students.

### Teaching staff

As previously indicated, the program teaching staff comprises five staff members, four general psychiatrists, and one clinical psychologist, and is complemented as needed by staff from other areas of the Faculty of Medicine and the University. The department also collaborates with specialists regionally and globally to ensure that students receive the needed instruction and the needed subspecialties. In addition to their teaching duties, faculty members of the Department of Psychiatry provide clinical services at SPH. Recently, staff members participated in a professional development course offered by the university to enhance their instructional competence.

### Student orientation and support

At the beginning of the program, our residents undergo a one-week, faculty-wide orientation with all other MMed students, which includes presentations on academic policies and procedures and relevant support services. Residents also participate in a department-specific orientation that addresses the details of the psychiatry program, such as its structure, expectations, introduction to training sites, and program staff.

Each resident is assigned a supervisor who provides regular feedback on performance and progress and acts as the first point of contact when there are challenges. When students need support during the program, in addition to faculty members, the program leverages existing support systems such as the faculty-wide student support office and the university-wide counseling center.

### Program delivery

The program is delivered primarily through clinical apprenticeships in hospital and community settings. Other complementary learning events, such as lectures, student presentations, and research, are delivered virtually or face-to-face. Timely, frequent, constructive feedback is essential to the program implementation to ensure that students stay engaged and can achieve the intended learning outcomes.

### Ongoing quality assurance

Residents provide feedback during monthly departmental meetings (which include staff and residents), which the department uses to make changes. For instance, following residents’ feedback, we have changed the course sequencing and the mode of delivery for some modules. Other quality assurance measures include internal and external moderation of exams.

### Staying contextually relevant

To ensure that our curriculum addresses culture-specific factors, the first module (GME 624: Behavioral Science and Socio-cultural Psychiatry) introduces students to the psychological, social, and cultural factors relevant to the theory and practice of psychiatry. Students are also introduced to local cultural issues pertinent to the practice of psychiatry in Botswana. Throughout the program, students must consider cultural factors as they interact with their patients, eg, during history taking and case presentations.

### Training platform and infrastructure

The program is being delivered at four government facilities: Sbrana Psychiatric Hospital and Princess Marina Hospital, which are national referral hospitals, and Athlone Hospital and Deborah Retief Memorial Hospital, which are district hospitals. SPH is the department's primary teaching site for the residency program and the undergraduate psychiatry clerkship. Residents also rotate in community psychiatry, in which they go to a rural area to conduct home visits, facilitate community mental health education, and carry out a project relevant to the community's identified needs.

In addition to classrooms, faculty offices, and a UB internet network, SPH is resourced with a small library to ensure seamless access to information for students, teaching staff, and hospital staff. The library is an extension of the campus-based, well-resourced Library with up-to-date subscriptions to the major journals and reading materials in hard copy and digital formats required for the course. It has internet connectivity, a few computers, and two full-time librarians specifically responsible for the Department of Psychiatry. The librarians were involved in planning the program and were given a list of the required reading material and other library resources. The librarians participate in monthly departmental meetings and facilitate presentations on topics relevant to the use of the library in research and accessing learning materials.

The program also leverages existing technological platforms to facilitate elements of the program. Students and staff can access MS Teams, which comprise multiple applications, including a video conferencing tool for internal and external communication.

### Student recruitment, retention, and employability

Advertisements are made through various national media platforms each year. The number of applicants has ranged from 8 to 13 annually. The program has admitted five residents per year in the past five years based on the number of faculty available to provide adequate clinical and research supervision. Four of the five residents in the first cohort completed their training in 2023 and have been employed by the government.

### Regulatory compliance

The program (qualification and learning program) has been officially registered with the Botswana Qualifications Authority (BQA). The BQA is the custodian of the national credit and qualifications framework and coordinates the national educational quality assurance system. Additionally, the program has obtained provisional accreditation from the Botswana Health Professions Council (BHPC). The BHPC is the legislative licensure body for health professionals, whose primary objective is to promote the highest standard in healthcare practice in Botswana. This includes accreditation and monitoring of health professions programs and registration and monitoring of healthcare professionals.

## Evaluation phase

The evaluation phase focuses on assessing program effectiveness and refining the curriculum based on evaluation data and aligns with Kern's revision step^19^ and Fink's integration review.^20^ It involves ongoing internal (by the department) and comprehensive (by either the department or regulatory bodies) program evaluation at specified intervals.

The department's ongoing program evaluation involves reviewing progression rates, summative and formative performance, dissertation progress, and completion rates. In addition to the feedback provided by residents through departmental meetings, as previously mentioned, rotation supervisors and coordinators meet with residents twice a semester to discuss their progress. Furthermore, residents provide formal feedback through the yearly evaluations of teaching staff and courses. Such evaluations guide and inform design, structure, curriculum, and resource changes to help improve the program.

As per UB policy, a comprehensive program review should occur soon after the first cohort graduates. The review will assess whether the program is achieving its broad vision of training contextually relevant psychiatrists and whether the graduates have achieved the intended program learning outcomes. This comprehensive review will draw from Kirkpatrick^40^ to assess the achievement of learning outcomes and Stufflebeam's Context, Input, Process, and Product (CIPP) model^41^ to ensure that we address the process and context. The findings will be shared with relevant stakeholders and used to take the necessary remedial action to ensure that the program stays responsive to contemporary teaching, learning, and assessment modalities.

## Discussion

This paper reflects on and describes the development of a psychiatry residency program in Botswana and contributes a process grounded in educational program development models and educational theories that others can utilize. We broaden the current literature of existing African psychiatry programs by sharing the underlying theories that undergird our teaching and our program learning outcomes and structure for others looking to start their own programs. In this section, we present our discussion as valuable insights and lessons learned: building collaborations to fill the gaps, utilizing technology to have a wider reach of expertise, leveraging institutional resources and processes, building pedagogical knowledge as a department, and increasing locally relevant research.

### Build collaborations to fill the gaps

Collaborations have been used effectively in Liberia,^15,16^ Somaliland,^17^ and Ethiopia^18^ to augment and extend the expertise of the local teaching team. For instance, in Liberia,^16^ US faculty were described as supplementing the “supervisory and teaching responsibilities of the two Liberia-based psychiatry faculty” (5). In Liberia, both intercontinental and intracontinental collaborations were leveraged to support the local psychiatry program. Similarly, we found intercontinental (University of Pennsylvania, USA), intracontinental (University of the Witwatersrand, University of KwaZulu Natal, both in South Africa), and local collaboration invaluable. Since the psychiatry department comprised only five general psychiatrists at the outset, it became imperative to enlist sub-specialists from local and international sources, particularly for the latter part of the program. We leveraged existing partnerships and built new ones, including individuals appointed as adjunct lecturers or visiting scholars. This approach did not necessitate a significant increase in financial expenditure but provided support, such as co-teaching and mentoring. The collaborations also helped stem the challenges of recruitment and retention^26^ that the medical school was initially experiencing.

### Utilize technology to have a wider reach of expertise

Investment in appropriate technology, including videoconferencing and reliable internet access, is critical in leveraging collaborations to support residency programs in LMICs. For instance, there was an initial investment in technology to support communication and access to digital resources in Liberia.^15,16^ In our case, we built on and expanded the existing University technological infrastructure to ensure connectivity at the national psychiatric hospital.^5,25^ Harnessing technology has enabled remote teaching by partners outside the country. For instance, our residents have joint monthly virtual sessions with their counterparts from the University of Kwa-Zulu Natal in South Africa. Locally, departments in the University, like the Department of Biomedical Sciences, Social Sciences, and Psychology, also teach the appropriate courses in the program. Since international bodies such as the World Psychiatry Association, The American Psychiatric Association, and The Royal College of Psychiatrists host many courses and resources,^42^ reliable internet access allows teachers and residents to access such resources. Besides internet access and video conferencing technology, the University invested in an extensive physical and digital collection for the medical school and the residency program when it was ultimately started.

### Leverage institutional resources and processes

The teaching and learning infrastructure (existing hospitals, furnished classrooms, internet access, library resources) already established for the undergraduate program meant we did not start from scratch when onboarding the graduate training. As such, there was no need for a significant capital outlay on infrastructure development. Additionally, there were established curriculum development and approval processes for University programs, including templates and guidelines, making the process easier. We also learned from the experiences of other residency programs that had started before us who had already experienced teething problems. While being part of an existing university has benefits, it can also present challenges when systems and processes lack the agility needed to accommodate the uniqueness of medical education.

### Build pedagogical knowledge as a department

Comprehensive pedagogical knowledge encompassing teaching, learning, assessment practices, and curriculum development processes is imperative for the effective development and implementation of programs.^43^ Since the program's inception, we have individually and collectively availed ourselves of diverse learning opportunities in this realm. Recently, we participated in a university-wide course facilitated by the Department of Medical Education to garner deeper insights into teaching and assessment practices. The hands-on course focused on reflective practice,^44,45^ coaching,^46,47^ self-direction, and self-regulation,^48–50^ and addressed a broad spectrum of teaching competence topics, including lesson planning, peer observation, personal philosophy of teaching, learning, and assessment, developing assessment tools and items, moderating assessment, reporting on assessment and courses, developing courses, writing learning outcomes, constructive alignment, blueprinting, and teaching methods. We believe that growing together pedagogically enables us to have a common language and understanding regarding learning, teaching, and assessment.

### Increase locally relevant research

Boyer (1990)^51^ has argued that the scholarship of discovery, commonly called research, is one of the four functions of the professorate, one that overlaps with other forms of scholarship—application, integration, and teaching.^51^ In addition to learning from our partners during the department's early days, we have continued to take advantage of many opportunities, including those at the University, to improve our scholarship. Since the establishment of the department, locally relevant mental health research has increased substantially. This increase is primarily due to research conducted by staff and students, which has helped fill the gap identified by stakeholders during the SWOT analysis during the planning stage of the program. This makes it easy for instructors to give examples that students can easily relate to and helps in increasing the evidence base for policymakers.

### Persist in the slow process of transforming psychiatric care in the nation

Academic medicine is not always consistent with the prevailing culture within which the program is housed. While multidisciplinary care is the best practice in psychiatric services,^52^ the prevailing culture in our context has been that of parallel service provision, where healthcare professionals attend to patients without a common goal, leading to poor patient outcomes. This is compounded by the resistance to continuing professional development among healthcare workers, as it is often seen as an exercise for academics and taking time away from pushing patient loads. Additionally, the use of contemporary evidence-based tools such as electroconvulsive therapy and vagal nerve stimulators may not be available and acceptable. As such, there is a need for the academic department to continually sensitize colleagues to the benefits of a multidisciplinary approach to resident training, the use of contemporary evidence-based service provision, and continuing professional development to improve patient outcomes.

## Conclusion

Botswana's experience adds to the literature showing that low- and middle-income countries can develop homegrown residency programs in psychiatry despite limited resources. These innovative approaches include leveraging existing university infrastructure and resources, harnessing the potential of technology, and collaborating with other established programs for mentoring, co-teaching, and clinical care. We are optimistic that this program will continue to succeed and lead to an adequate pool of homegrown psychiatrists who will stay and work in the country and help address the human resources shortage.
